# Combinatorial measurement of critical cooling rates in aluminum-base metallic glass forming alloys

**DOI:** 10.1038/s41598-021-83384-w

**Published:** 2021-02-16

**Authors:** Naijia Liu, Tianxing Ma, Chaoqun Liao, Guannan Liu, Rodrigo Miguel Ojeda Mota, Jingbei Liu, Sungwoo Sohn, Sebastian Kube, Shaofan Zhao, Jonathan P. Singer, Jan Schroers

**Affiliations:** 1 Department of Mechanical Engineering and Materials Science, Yale University, New Haven, CT 06511 USA; 2 Department of Mechanical and Aerospace Engineering, Rutgers, The State University of New Jersey, Piscataway, NJ 08854 USA; 3 Qian Xuesen Laboratory of Space Technology, Beijing, 100094 China; 4 College of Mechanical and Electrical Engineering, Beijing University of Chemical Technology, Beijing, 100029 China

**Keywords:** Characterization and analytical techniques, Design, synthesis and processing, Glasses, Metals and alloys

## Abstract

Direct measurement of critical cooling rates has been challenging and only determined for a minute fraction of the reported metallic glass forming alloys. Here, we report a method that directly measures critical cooling rate of thin film metallic glass forming alloys in a combinatorial fashion. Based on a universal heating architecture using indirect laser heating and a microstructure analysis this method offers itself as a rapid screening technique to quantify glass forming ability. We use this method to identify glass forming alloys and study the composition effect on the critical cooling rate in the Al–Ni–Ge system where we identified Al_51_Ge_35_Ni_14_ as the best glass forming composition with a critical cooling rate of 10^4^ K/s.

## Introduction

Since their discovery^1^, a large number of alloys have been reported to form metallic glasses and some even bulk metallic glasses (BMGs) which require cooling rates below 1000 K/s^2,3^. However, it has been estimated that only about 10% of the composition space of potential BMG formation has been considered thus far^4^. The ability of an alloy to form a glass, so called glass forming ability (GFA), is quantified by the critical cooling rate, *R*_c_. *R*_c_ is the lowest rate a liquid can be cooled to avoid crystallization and vitrify into a glass^5^. Direct measurement of *R*_c_ has been cumbersome and challenging and therefore, has only be determined for a few alloys^6–13^. Instead, other more accessible properties have been measured to approximate glass forming ability^14,15^. This includes critical casting thickness, reduced glass transition temperature *T*_rg_ = *T*_g_/*T*_l_^16^ and derivatives and extensions of *T*_rg_^17–19^.

To address the large potential compositional space more effectively, combinatorial approaches have been used for the fabrication of large number of alloys in thin film alloy libraries^20–27^ and high-throughput methods developed to provide information about glass formation^22,27,28^. However, attempts to directly quantify critical cooling rates of alloys in such libraries have been limited to the as-sputtered state which forms under a cooling rate exceeding 10^8^ K/s^29,30^. Such high cooling rates are not comparable with cooling rates for practical BMGs of ~ 10^3^ K/s. Some progress was made recently by reheating the thin films and reducing the cooling rate to ~ 10^5^ K/s^28^, however still no significant variations of the cooling rate could be achieved, a requirement to determine the critical cooling rate. Direct measurement of critical cooling rates of thin film alloys has been recently suggested through nano calorimetry^31–33^. This method can be extended into a combinatorial method; however, the fabrication of the sensors is sophisticated and cumbersome, hence to date limited.

Here, we report a fast screening method which is based on laser heating to directly measure *R*_c_ in thin film alloy libraries. Based on Single Pulse Laser Annealing, cooling rates ranging from 10^2^ to 10^6^ K/s can be realized during solidification of the alloys. As an example, we determined *R*_c_ for a large number of alloys in the Al–Ge–Ni system, and identified the best glass forming composition as Al_51_Ge_35_Ni_14_ with a critical cooling rate of 10^4^ K/s.

## Results

**Figure 1:**
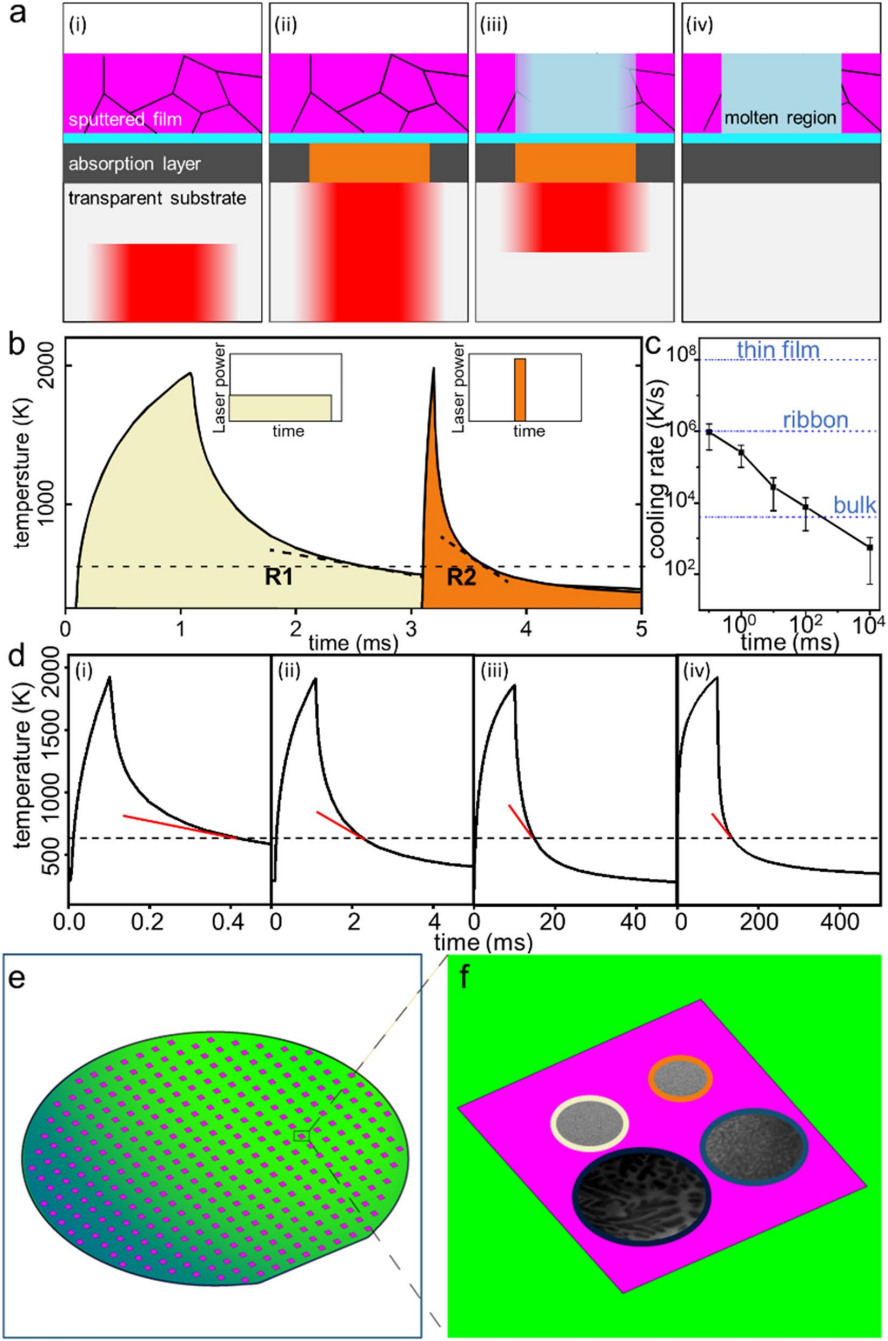
Experimental setup of single pulse laser annealing. (**a**) Schematics of single pulse laser annealing. Laser beam (red) passes transparent sapphire substrate (light gray) and heats tungsten absorption layer ((i) and (ii)). Heat generated from absorption layer then dissipates across a 10 nm dielectric layer and melt the thin film sample (iii). After Laser beam is turned off, system is cooled down by natural cooling through sapphire substrate, and thin film sample solidifies (iv). (**b**) in Single Pulse Laser Annealing, thermal profile is controlled by varying the duration time of the laser pulse. Thermal simulation of two laser pulses with duration time of 0.1 ms (orange) and 1 ms (yellow) are shown with significant varied cooling rate (R1 and R2). (**c**) By changing the laser pulse duration time, from 0.1 ms to 10 s , cooling rates ranging from 10^6^ to 10^3^ K/s can be realized. Error bar is defined by cooling rates at temperatures between 500 and 800 K, which is the typical region of the nose temperature of TTT diagram for most glass forming alloys. (**d**) Simulated result of laser heating/cooling profile with different laser duration time: (i) 0.1 ms, (ii) 1 ms, (iii) 10 ms, and (iv) 100 ms, showing cooling rates controlled over orders of magnitude. (**e**) High-throughput measurement on material library. Sputtered material library is meshed into ~ 200 different compositions. (**f**) Four different laser pulses are applied to each composition, giving cooling rates varying through 4 orders of magnitude.

The experimental setup comprises of the universal thin-film heating architecture, library synthesis, and a scanning system (Fig. 1). For the universal thin-film heating architecture we use Single-Pulse Laser Annealing to locally heat and melt a thin film alloy. A sapphire (Al_2_O_3_) wafer is used as a laser transparent substrate. Instead of relying on absorption of the laser by the alloy, we use an absorption layer made from tungsten, 100 nm thick, which is located above the sapphire wafer. Calculated transmission rate through the tungsten layer based on its refractive index is about 1%^34^. To decouple the absorption layer from the alloy, a 10 nm dielectric separation layer of Al_2_O_3_ is deposited on the tungsten. Subsequently, the alloys, as alloy libraries, are sputtered on the structure. For efficient energy absorption of the tungsten layer, we use a 1070 nm and 200 W diode laser as heating source which warrants an absorption of ~ 60%, as calculated from boundary equations of electromagnetic waves propagating through multilayer media^34^ (Fig. 1a(ii)). Within this heating architecture, the alloy is heated not directly through the laser but through heat conduction from the tungsten absorption layer. The advantage of such design is that heating, maximum temperature, and cooling rate are essentially independent from the specifics of the sample alloy and its absorption coefficient. Instead they are defined by the absorption layer thickness and laser pulse settings, which can be kept constant and hence a controlled, calibrated, and predictable heating and cooling profile can be generated. Therefore, proposed universal thin-film heating architecture allows us to pre-determine and/or simulate the heating and cooling rates and control them by the laser setting, independent of the specifics of the alloy within the alloy library. This allows to apply a priori known heating and cooling rates over alloy libraries of largely varying chemical composition.

To quantify the cooling rates, we use simulations to solve the heat flow equation numerically (see supplementary materials). A 3D model based on the universal thin-film heating architecture was imported into COMSOL. Heat generation in the simulation originates from the interaction between the tungsten layer and the laser beam. Heat can dissipate through the sample and also through the sapphire. The temperature profile of the thin film region from this model was subsequently probed and used to calculate the cooling rate for the Single Pulse Laser Annealing.

Cooling rates are controlled through laser pulse length and peak power (Fig. 1b). In general, a long and low power pulse leads to a low cooling rate and a short, high-power pulse to a high cooling rate. The cooling rates for all laser pulse profiles vary as a function of temperature. For the here considered Al–Ni–Ge alloys, the critical temperature range to avoid crystallization is ~ 600 K. This temperature is assumed at the nose of the time–temperature–transformation curve, which has been previously observed to be located approximately at (*T*_*l*_ + *T*_*g*_)/2^12,35–38^. Throughout the document, the cooling rates are calculated at 600 K. Utilizing the simulations with specific heating conditions where the pulse length is varied from 0.1 ms to 10 s and the peak power is modified accordingly to maintain a constant peak temperature, cooling rates can be varied from 10^2^ to 10^6^ K/s (Fig. 1c).

The alloy library is synthesized through combinatorial sputtering from three sputtering guns. These guns are aligned in a tetrahedral geometry to deposit varying quantities of the alloy’s elements as a function of the x–y position on the substrate^22^. Specifically, we used Al, Ni, and Ge and sputtered a composition region covering 30–83 at.%, 4–28 at.% Ni, and 11–63 at.% Ge. Compositions were measured by energy dispersive X-ray spectroscopy (EDS). The alloy library is separated into ~ 200 individual patches, each one 2 mm in diameter and separated by 5 mm. As sputtered films are ~ 500 nm thick.

The universal thin film heating system is positioned in a vacuum chamber and operates under vacuum conditions of 10^–3^ mTorr. A computer-controlled X–Y stage, which is synchronized with the laser controller, moves the alloy library between alloy patches (Fig. 1e) and within a patch to four locations that are sufficiently far separated to prevent cross patch interference (Fig. 1f). For the four locations on one alloy patch, the laser pulses vary to result in cooling rates of about 10^6^, 10^5^, 10^4^, and 10^3^ K/s, respectively. Application of each laser pulse and subsequent relocation to the next location takes 10 s. Hence, it takes ~ 2.5 h to apply four different rates to all alloys in a library of 200 alloys.

We carried out critical cooling rate measurement with Single Pulse Laser Annealing on Al–Ge–Ni alloy library. Alloys are separated by approximately 2 at.% Al, 1 at.% Ge, and 0.3 at.% Ni. For each alloy we applied four cooling rates of $1\times {10}^{6}$ K/s, $2.5\times {10}^{5}$ K/s, $1.8\times {10}^{4}$ K/s, and $4.2\times {10}^{3}$ K/s.

**Figure 2:**
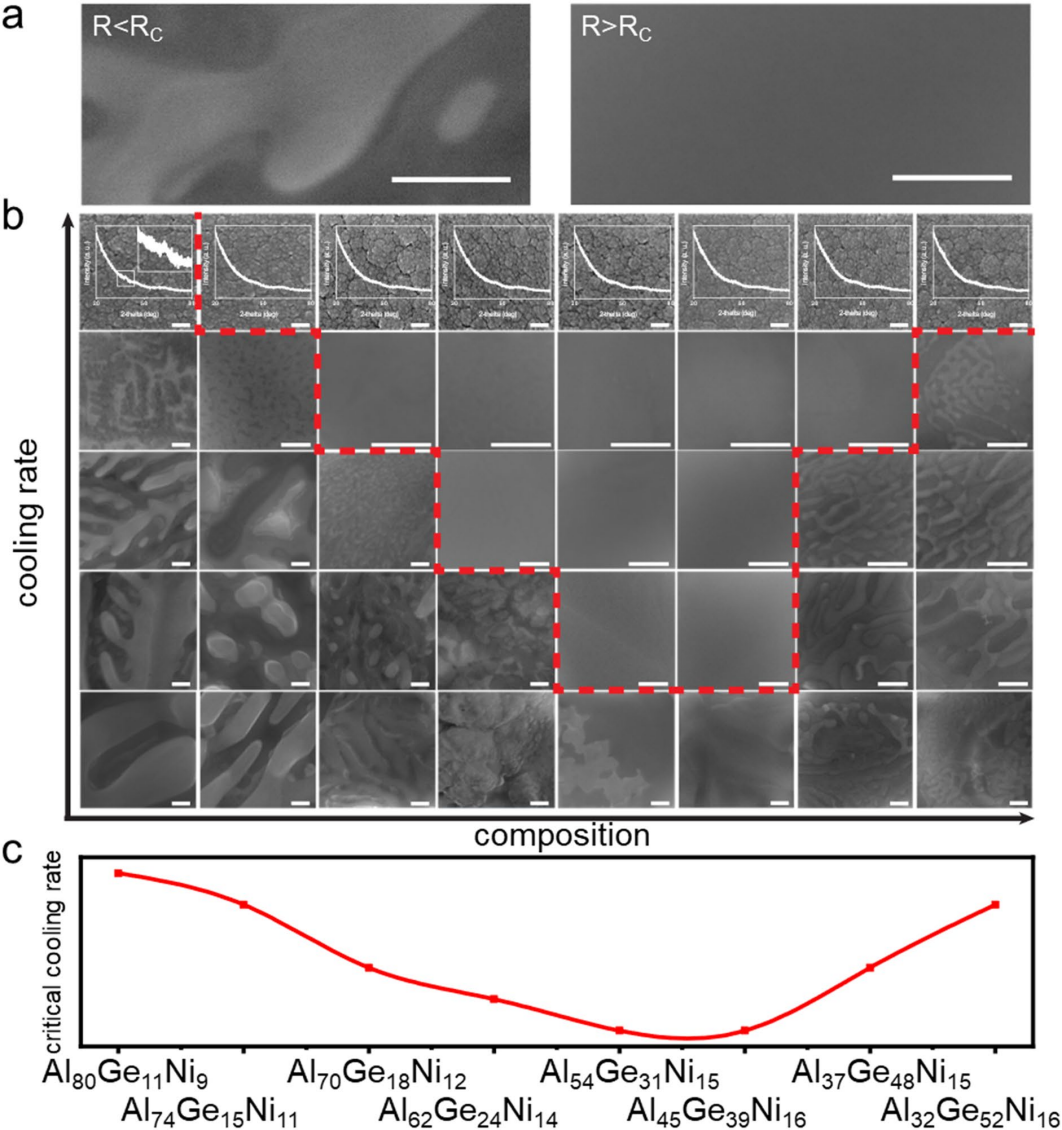
Microstructure analysis to reveal glass formation and critical cooling rate. (**a**) Characteristic microstructure for a crystalline alloy, *R* < *R*_c_ and an alloy that vitrified and formed glass, *R* > *R*_c_ (scale bar: 100 nm). (**b**) Microstructure mapping of Al_80_Ge_11_Ni_9_, Al_74_Ge_15_Ni_11_, Al_70_Ge_18_Ni_12_, Al_62_Ge_24_Ni_14_, Al_54_Ge_31_Ni_15_, Al_45_Ge_39_Ni_16_, Al_37_Ge_48_Ni_15_, and Al_32_Ge_52_Ni_16_ (from left to right). This composition line is indicated in Fig. 3 as the white dashed line. Each column represents microstructures of one alloy for different cooling rates. The first row shows result of as-sputtered film, representing the highest cooling rate of ~ 10^8^ K/s (with XRD curve superimposed). From the second row to the fifth row results from Single Pulse Laser Annealing of different cooling rates, in sequence, $1\times {10}^{6}$ K/s, $2.5\times {10}^{5}$ K/s, $1.8\times {10}^{4}$ K/s, and $4.2\times {10}^{3}$ K/s are shown (scale bars: 100 nm). (**c**) Measured critical cooling rates which are based on the microstructure characterization of the eight compositions in (**b**).

**Figure 3:**
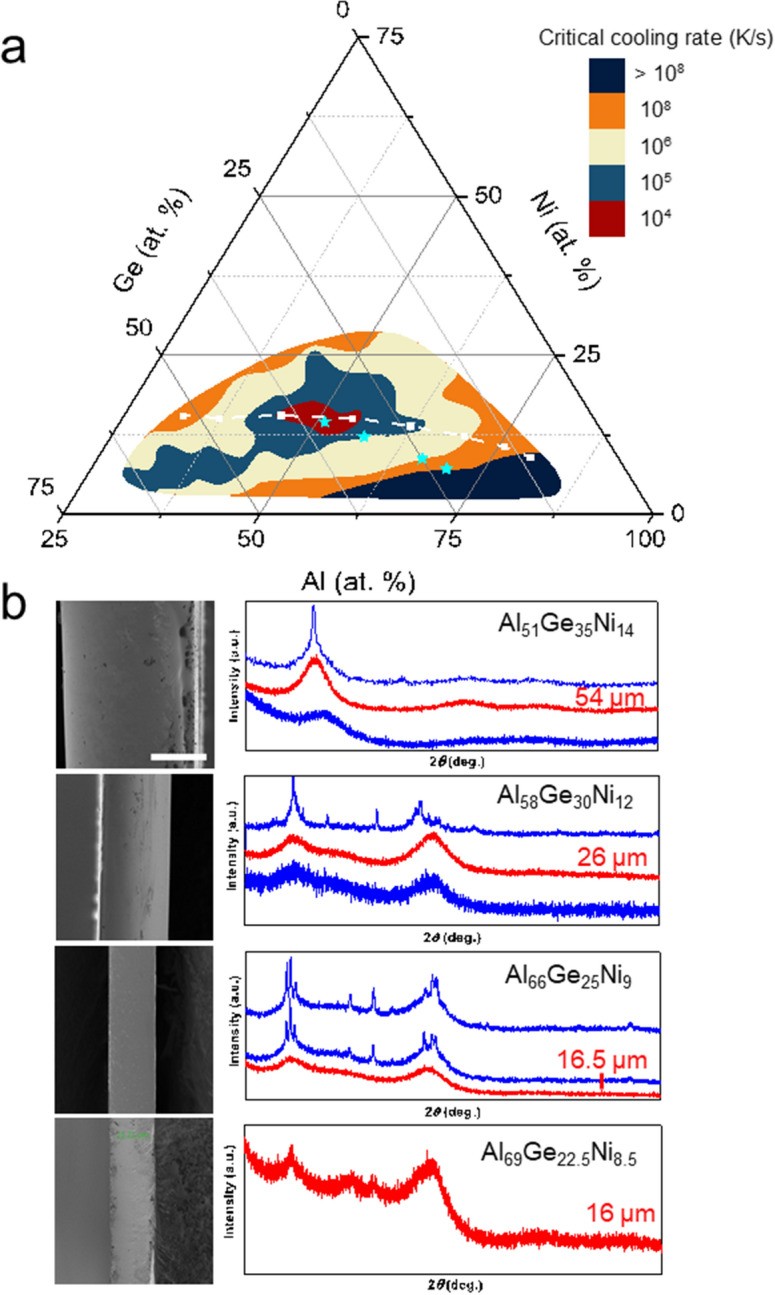
Critical cooling rate mapping and results from melt spinning samples. (**a**) Summary of critical cooling rates of the considered Al–Ni–Ge alloys spanning 6 orders of magnitude. The white dash line labels the compositions shown in Fig. 2. (**b**) Measured *R*_*c*_ was further revealed with melt spinning samples. For 4 different compositions marked as stars in (**a**), melt spinning samples with decreasing rotation speeds were made and glass to crystalline transition was revealed by XRD. With decreasing rotation speed giving thicker sample thickness, critical thickness of each composition is revealed. XRD curves from these thicker to thinner samples are shown from top to bottom of each composition. Critical thickness was measured by SEM from amorphous sample of the largest thickness, with corresponding XRD curves shown in red. XRD curves of samples either above or below critical thickness are shown in blue. Scale bar: 20 μm.

The characterization to evaluate *R*_c_ is based on microstructure analysis using scanning electron microscopy (SEM). Specifically, we distinguish between crystalline microstructure and amorphous microstructure (Fig. 2). If an alloys’ microstructure under specific cooling condition reveals a contrast, which is indicative of a crystalline structure, we conclude that applied cooling rate is smaller than *R*_c_. If the alloy and cooling conditions reveal a homogenous amorphous microstructure, we conclude that applied cooling rate is larger than *R*_c_ (Fig. 2a). This allows to determine *R*_c_ as long as it is in the range of cooling rates of 10^2^–10^6^ K/s which are achievable within this method. Example microstructures for different alloys (the dashed line from Al_80_Ge_11_Ni_9_ to Al_32_Ge_52_Ni_16_ in Fig. 3a) and various cooling rates are shown in Fig. 2b. Structural characterization through transmission electron microscopy (TEM) have been attempted but abandoned due challenges originating from the low activation energy of nucleation and low melting temperatures in Al-based metallic glasses^39,40^.

We used this technique which is exemplified in Fig. 2 to determine *R*_c_ for all ~ 200 Al–Ge–Ni alloys (Fig. 3). On as-sputtered film, large composition regions form an amorphous structure. Only at the aluminum rich corner the cooling rate during sputtering of ~ 10^8^ K/s is insufficient to suppress crystallization (labeled as dark blue). All the compositions that form an amorphous phase at rate > 10^8^ K/s are also exposed through laser spike annealing treatment to lower rates and subsequent characterization. As the cooling rate decreases, the glass forming composition region shrinks rapidly (Fig. 3a). Specifically, we indicated cooling rates of 10^8^ K/s as orange, 10^6^ K/s as light yellow, 10^5^ K/s as blue, and 10^4^ K/s as red, which points at Al_51_Ge_35_Ni_14_ as the composition with the best glass forming ability in the Al–Ge–Ni system.

To verify the identified glass forming alloys in this system and the variations in GFA that we determined through Single Pulse Laser Annealing, we fabricated four selective alloys through melt spinning (marked in Fig. 3a as stars). These alloys are Al_51_Ge_35_Ni_14_ (*R*_c_ ~ 10^4^ K/s), Al_58_Ge_30_Ni_12_ (*R*_c_ ~ 10^5^ K/s), Al_66_Ge_25_Ni_9_ (*R*_c_ ~ 10^6^ K/s), and Al_69_Ge_24_Ni_7_ (*R*_c_ ~ 10^8^ K/s). To vary the cooling rate during melt spinning we used a range of rotation speeds for each alloy which allows us to determine a critical casting thickness, *d*_c_. We measured for Al_69_Ge_24_Ni_7_ a critical casting thickness of *d*_c_ < 16 μm, for Al_66_Ge_25_Ni_9_ *d*_c_ = 16.5 μm, for Al_58_Ge_30_Ni_12_ *d*_c_ = 26 μm, and for Al_51_Ge_35_Ni_14_ *d*_c_ = 54 μm (Fig. 3b). Both techniques, melts spinning and Single Pulse Laser Annealing, reveal the same trends of *R*_c_ or *d*_c_ with composition.

The absolute values of *R*_c_ can be translated into *D*_*c*_ through *R*_c_ = 1000/*D*_*c*_^2^ (*D*_c_ in mm)^41^. The latter correlation assumes an absence of a thermal resistance of the interface, and an infinite thermal conductivity and thermal mass of the coolant^41^. This gives *R*_*c*_ > $4\times {10}^{6} \mathrm{K}/\mathrm{s}$ for Al_69_Ge_8.5_Ni_22.5_, *R*_*c*_ = $3.7\times {10}^{6} \mathrm{K}/\mathrm{s}$ for Al_66_Ge_9_Ni_25_, *R*_*c*_ = $1.3\times {10}^{6} \mathrm{K}/\mathrm{s}$ for Al_58_Ge_30_Ni_12_ and *R*_*c*_ = $3\times {10}^{5} \mathrm{K}/\mathrm{s}$ for Al_51_Ge_35_Ni_14_. The *R*_*c*_ values from melt spinning and Single Pulse Laser Annealing are following the same trends with alloy composition. The lower absolute values in the melt spun ribbon compare to the ones measured by Single Pulse Laser Annealing may originate from the high strain rates during solidification during melt spinning which have been reported to accelerate crystallization^41, 42^.

## Discussion

Al-based metallic glasses are an important family of glass forming alloys with tremendous potential technical importance^43^. They generally exhibit high specific strength, high corrosion resistance, which are paired with other attractive attributes^43,44^. Their glass forming ability (GFA), however, is generally low, with largest reported critical casting thickness of ~ 1 mm^45^. Different from other glass forming systems, the composition of Al-based glasses are generally not located at deep eutectics, which makes the discovery of Al-based metallic glasses particularly challenging^46,47^.

Typically the GFA within one alloy system changes rapidly with alloy composition^48^. This suggest that large composition regions have to be studied at a fine grid to discover alloys with highest GFA within one system. It is also important to notice that only limited data exist on GFA as a function of composition spanning larger ranges. Such data will be important to further understand glass formation, particularly when combined with other data, e.g. viscosity^17^.

We found here that for Al–Ge–Ni alloys, the effect of alloy content on GFA is the highest for Ni where *R*_*c*_ changes by one order of magnitude per 3 at.%. This larger sensitivity of Ni content compare to the Ge content suggest that the effect of atomic size ratio and their corresponding fractions is critical^49,50^ and not predominately the ratio of metal to metalloid fraction^51^ as in other non-Al-based glass forming alloys such as Au–Cu–Si^30^.

**Figure 4:**
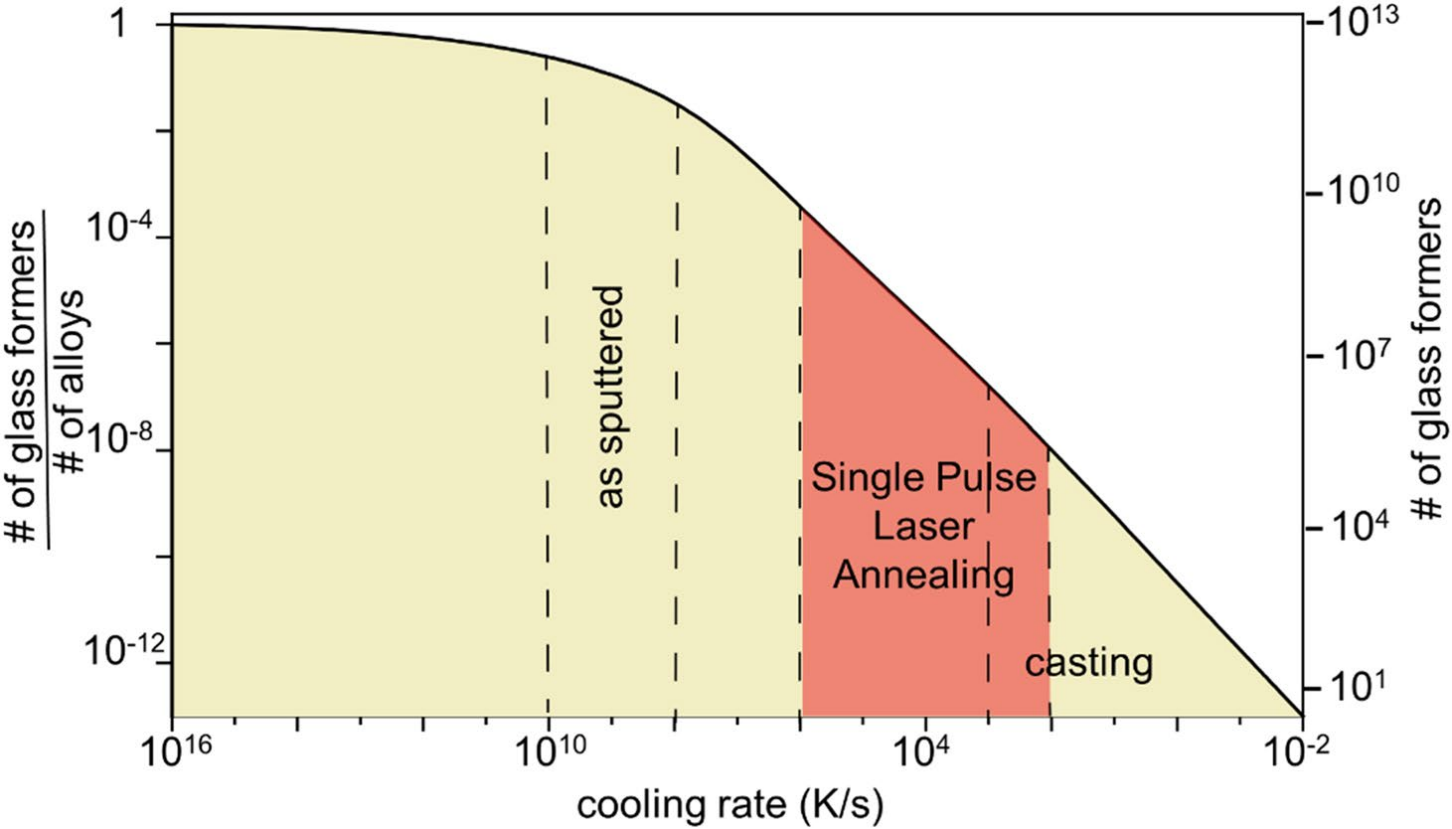
Order of magnitude estimation of the potential number of glass formers as fractions of the total number of alloys for different cooling rates. Data is estimated for quinary systems including all the lower order systems from 32 glass forming elements^4^. For cooling rates above ~ 10^16^ K/s all alloys and even elemental metals form glasses^52^. When decreasing the cooling rate to ~ 10^9^ K/s a large fraction, ~ 50% form glasses^29,53,54^. For cooling rates around 10^3^ K/s it was estimated that 10^6^ alloys are potential bulk metallic glass formers. For low cooling rates below 10^–2^ K/s no glasses are formed^7,55^. The cooling rates accessible with Single Pulse Laser Annealing span a broad band and allow to effectively identify best glass formers with introduced approach.

We will now discuss the efficiency of the here described method to identify best glass formers in an alloy system. For this we estimate the relative fraction of glass formers as a function of cooling rate. We assume that 32 elements from the periodic table have been considered in glass forming alloys^4^. For these elements, we estimate the numbers and fractions of glass formers through their combinations where we consider up to quinary systems (Fig. 4) as it is discussed in detail in reference^4^. For cooling rates above ~ 10^16^ K/s all alloys and even elemental metals form glasses^52^. When decreasing the cooling rate to ~ 10^9^ K/s a large fraction, ~ 50% has been observed to form glasses^29,53,54^. For cooling rates around 10^3^ K/s it had been estimated, based on extrapolations from experimental results that 10^6^ alloys are potential bulk metallic glass formers^4^. For low cooling rates below 10^–2^ K/s no glasses are formed^7, 55^. These data provide the basic for the estimation in Fig. 4 and allow us to discuss alloy development strategies for metallic glass formers. Figure 4 reveals that characterizing alloys for their glass formation during sputtering is not very insightful as these identified potential glasses have a very low statistical probability of 2/10^6^ to form bulk metallic glasses. For an effective identification of best glass formers in an alloy system it is required to (1) characterize glass formation for a broader range of cooling rates (2) this range covers high cooling rates where the probability is high for glass formation and low cooling rates to distinguish between the glass formers and identify best glass former in alloy system, and (3) fabricate and characterize fast. Such requirements are realized with the here introduced Single Pulse Laser Annealing method spanning the range of cooling rates from 10^2^ to 10^6^ K/s. In particular with feedback from higher cooling rates scanning results, where a larger composition space forms glasses. One can then down select alloys for further exposure to a lower cooling rate and repeat this step sequentially which provides a highly effective method to identity best glass formers in an alloy system. This approach, which is motivated by the considerations that are summarized in Fig. 4 are key for an effective alloy development approach. For example, in conventional alloy development when bulk samples are used, the statistical probability to identify a glass is on the order of 1/10^7^. The fact that this bulk process is very slow and that glass forming ability changes rapidly with composition combined with the low probability of an alloy to form BMGs explains why only very few of the potential BMG forming alloys have been discovered thus far. That the number is higher, ~ 1/10^6^ than suggested by statistical probability, 1/10^7^ is a result of using “guidelines” such as Inoue’s rules^3^, and compositions close to a eutectic composition^56^ that enhances discovery probabilities. The range of cooling rates that can be realized during Single Pulse Laser Annealing method spanning the range of cooling rates from 10^2^ to 10^6^ K/s. Here, particularly when using a range of cooling rates, from high to low, provides a much more effective strategy to identify BMGs.

In conclusion, we report a novel method to rapidly measure critical cooling rates of glass forming alloys through a large cooling rate range based on Single Pulse Laser Annealing. A universal thin film heating architecture was designed to establish identical cooling profiles which can be varied over thin film sample with different compositions and laser absorption rates. With this novel method, we studied Al–Ge–Ni, which exhibit critical cooling rates that are difficult to determine with other experimental techniques. We directly measure *R*_*c*_ over a large composition space which reveals the GFA profile and further allows us to identify best glass forming alloy in this system. We expect, by closing the gap between characterization techniques on thin film glass formation and bulk glass formation through here introduced Single Pulse Laser Annealing, a rapid growth in BMG discovery rate.

## Methods

### Preparation of uniform heating substrate and material library

The uniform heating substrate was fabricated based on a 600 μm thick 4-in. sapphire wafer (C-plane, double sides polished). An absorption layer, 100 nm Tungsten, was deposited on the sapphire wafer by co-sputtering. After that the wafer was taken to an atomic layer deposition (ALD) system, where a 10 nm Al_2_O_3_ dielectric layer was grown upon Tungsten. 500 nm thick material library of Al–Ge–Ni was deposited on the pre-prepared uniform heating substrate by co-sputtering. The base pressure was lower than 10^–6^ Pa and working pressure 0.3 Pa. High purity (99.99%) material targets, Al, Ge and Ni, were sputtered with a tilting angle of 29.8° towards the substrate to create the composition gradient across the wafer. As sputtered library was characterized by X-ray diffraction (XRD) to reveal atomic structures under high cooling rate by sputtering. For composition measurement on the material library, to avoid extra signal from underlying Al_2_O_3_ and sapphire (Al containing), the same material library (300 nm) was deposited on a 4-in. Si wafer for EDS measurement, when fabricating which all sputtering conditions including pressure, power and tilting angle were carefully controlled to be the same as used for the material library deposited on uniform heating substrate.

### Method of thermal simulation

The thermal simulation is conducted using COMSOL Multiphysics® thermal finite element analysis in an axisymmetric configuration. The heating substrate is modeled as 100 nm of tungsten coated on top of a 600 µm sapphire substrate. The thermal properties of aluminum are used to represent the properties of the MG layer. All thermal properties are simplified to be thermally independent. The whole wafer is assumed to be isotropic with an initial uniform temperature at 293.15 K. The periphery is set to a constant temperature boundary condition at 293.15 K, while the other boundaries are set to the insulation boundary condition. The size of the domain is selected to not affect the simulation result by systematic expansion. The laser heating is modeled as a temporal rectangle pulse and spatial Gaussian surface heat source using an experimentally-measured absorbance of 0.367.

## Supplementary Information

Supplementary Informations.
