# Supplementary material for: Combinatorial measurement of critical cooling rates in aluminum-base metallic glass forming alloys
Source: Sci Rep. 2021 Feb 16;11:3903. doi: 10.1038/s41598-021-83384-w (PMC7887254; doi:10.1038/s41598-021-83384-w)
Supplement: Supplementary file 1 — Supplementary Informations. [file 41598_2021_83384_MOESM1_ESM.docx]

Supplementary Information for

**Combinatorial Measurement of Critical Cooling Rate in Aluminum-base metallic glass forming alloys**

**Naijia Liu^1^, Tianxing Ma^2^, Chaoqun Liao^3,4^, Guannan Liu^1^, Rodrigo Miguel Ojeda^1^, Jingbei Liu^1^, Sungwoo Sohn^1^, Sebastian Kube^1^, Shaofan Zhao^3^, Jonathan Singer^2^ and Jan Schroers^1^ ***

***^1^ Department of Mechanical Engineering and Materials Science, Yale University, New Haven, CT 06511, USA.***

***^2^ Department of Mechanical and Aerospace Engineering, Rutgers, the State University of New Jersey, NJ 08854, USA.***

***^3^ Qian Xuesen Laboratory of Space Technology, Beijing 100094, China.***

***^4^ College of Mechanical and Electrical Engineering, Beijing University of Chemical Technology, Beijing 100029, China.***

**** Corresponds to: jan.schroers@yale.edu***


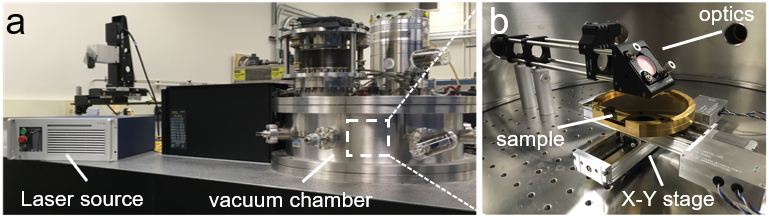


**Supplementary Figure 2.** Images of the laser scanning system. Laser is delivered through fiber into a high-vacuum chamber which contains sample and universal heating setup. A program-controlled X-Y linear stage is used to move wafer sample (with thin alloy film facing down) from point to point. An optical system is used to focus the laser beam and reflects it to heat the sample from sapphire side (backside).


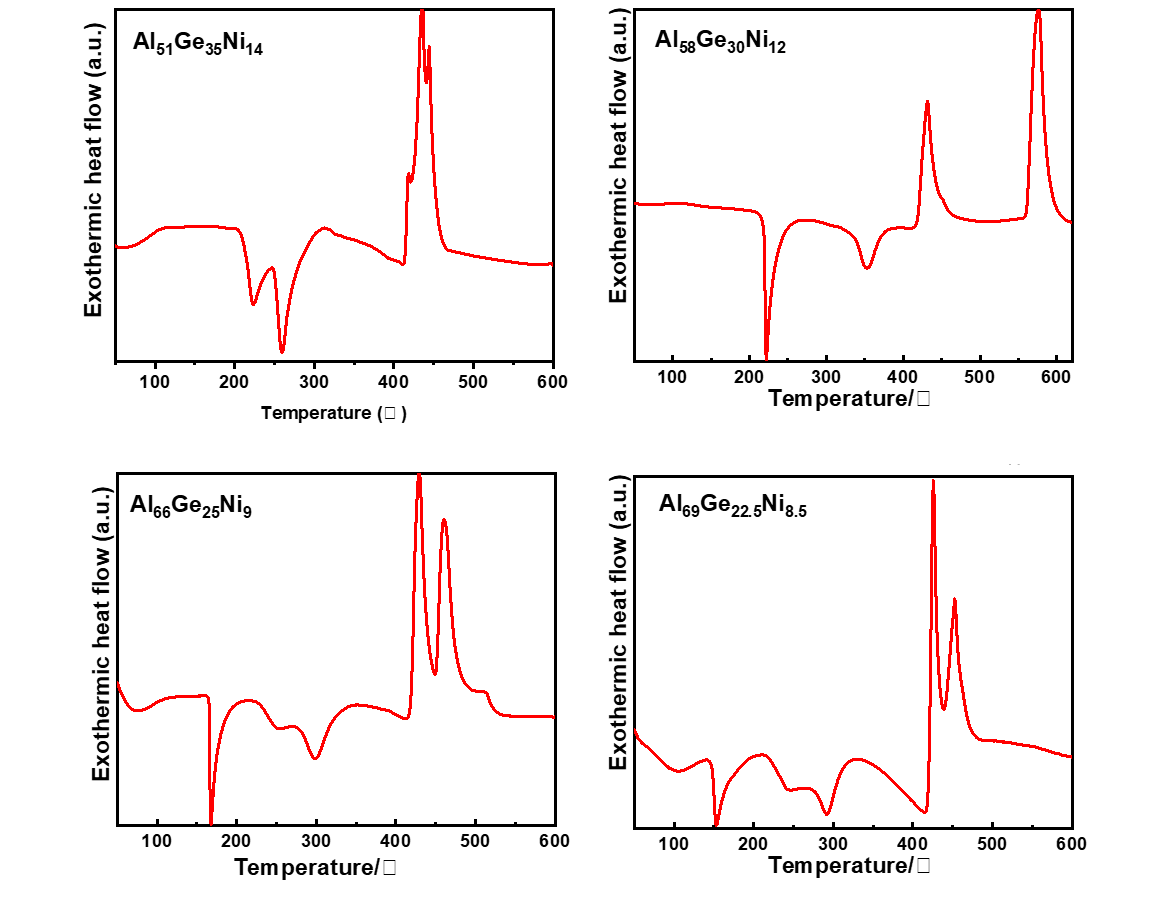


**Supplementary Figure 2.** DSC curves from melt spinning samples of the four selected compositions in Figure 4a. Heating rate: 20 K/s.
